# Aconitine induces cardiomyocyte damage by mitigating BNIP3‐dependent mitophagy and the TNFα‐NLRP3 signalling axis

**DOI:** 10.1111/cpr.12701

**Published:** 2019-10-27

**Authors:** Fu Peng, Nan Zhang, Chunting Wang, Xiaoyun Wang, Wei Huang, Cheng Peng, Gu He, Bo Han

**Affiliations:** ^1^ West China School of Pharmacy, State Key Laboratory of Biotherapy West China Hospital Sichuan University Chengdu China; ^2^ Key Laboratory of Southwestern Chinese Medicine Resources School of Pharmacy Chengdu University of Traditional Chinese Medicine Chengdu China

**Keywords:** aconitine, BNIP3, cardiomyocyte, mitophagy

## Abstract

**Objectives:**

Aconitine, the natural product extracted from *Aconitum* species, is widely used for the treatment of various diseases, including rheumatism, arthritis, bruises, fractures and pains. However, many studies have reported cardiotoxicity and neurotoxicity caused by aconitine, but the detailed mechanism underlying aconitine's effect on these processes remains unclear.

**Materials and methods:**

The effects of aconitine on the inflammation, apoptosis and viability of H9c2 rat cardiomyocytes were evaluated by flow cytometry, Western blot, RNA sequencing and bioinformatics analysis.

**Results:**

Aconitine suppressed cardiomyocyte proliferation and induced inflammation and apoptosis in a dose‐ and time‐dependent manner. These inflammatory damages could be reversed by a TNFα inhibitor and BNIP3‐mediated mitophagy. Consistent with the in vitro results, overexpression of BNIP3 in heart tissue partially suppressed the cardiotoxicity of aconitine by inhibiting apoptosis and the NLRP3 inflammasome.

**Conclusions:**

Our findings lay a foundation for the application of a TNFα inhibitor and BNIP3 to aconitine‐induced cardiac toxicity prevention and therapy, thereby demonstrating potential for further investigation.

## 1 INTRODUCTION

The *Aconitum* species are well known for their medicinal properties globally; clinical practice demonstrates that many species exhibit beneficial therapeutic actions in the treatment of rheumatism, arthritis, bruises, fractures, pains and other conditions.^1^ As many as 600 efficient traditional Chinese medicine (TCM) formulations comprising *Aconitum* species have been identified from both historical literature and modern clinical reports, such as “Fuzi Lizhong Borus” (a formulation for deficiency—cold in the spleen and stomach), “Jingui Shenqi Borus” (a formulation for warming and invigorating kidney yang) and “Shenfu Injection” (a formulation for preventing a person from collapsing by restoring yang).^1^

*Aconitum* species have been reviewed broadly on the basis of their phytochemical constituents and uses.^2^, ^3^, ^4^, ^5^, ^6^ The main chemical constituents of *Aconitum* plants are alkaloids, flavonoids, saponins, saccharides and fatty acids. *Aconitum* alkaloids, which comprise C19‐norditerpenoid alkaloids and C20‐norditerpenoid alkaloids, are the dominant ingredients responsible for the pharmacological activity and therapeutic efficacy of *Aconitum* species. As an indispensable alkaloid, aconitine plays an important role in the bioactivities of analgesic, diuretic, anti‐tumour, anti‐asthma and anti‐inflammatory agents.^7^, ^8^ However, the improper use of aconitine poses a high risk of severe poisoning. Cardiotoxicity and neurotoxicity are the main toxic effects of aconitine, and these are due to its effects on the voltage‐sensitive sodium channels of the cell membranes of excitable tissues, including the myocardium, nerves and muscles.^9^, ^10^, ^11^, ^12^ The typical symptoms of aconitine poisoning include palpitation, vomiting, nausea, arrhythmia, shock, dizziness, hypotension and coma.

Emerging studies showed that the cardiotoxic effects of aconitine most likely involve alterations of ion channels, energy metabolism and oxidative injury.^13^, ^14^, ^15^, ^16^, ^17^, ^18^ Nevertheless, the dominant molecular mechanism in signalling pathways underlying aconitine's cytotoxicity remains unclear. In the present study, aconitine induced apoptotic cell death of H9c2 cardiomyocytes by activating the TNFα and NLRP3 pathways. The combination of an apoptosis inhibitor and an NLRP3 inhibitor could reverse aconitine's cytotoxicity. The gene set enrichment analysis (GSEA) of RNA‐sequencing results suggests that the NLRs, apoptosis and autophagy pathways were significantly enriched in aconitine‐treated cardiomyocytes. Furthermore, mitochondrial damage and mitophagy inhibition were observed after incubation of aconitine with H9c2 cells. The introduction of a TNFα inhibitor or BNIP3 overexpression markedly reversed aconitine‐induced NLRP3 activation and mitophagy inhibition. The intramyocardial injection of BNIP3‐overexpression adenovirus significantly suppressed aconitine‐induced myocardial inflammatory fibrosis, apoptosis, and activation of the TNFα and NLRP3 signalling pathways. These findings might provide new insights into the molecular mechanisms underlying aconitine's cardiotoxicity and suggest novel strategies to alleviate aconitine‐induced myocardial injury.

## 2 MATERIALS AND METHODS

### 2.1 Reagents

The NLRP3 inhibitor MCC950, TNFα inhibitor Enbrel and aconitine were obtained from Shanghai Selleck Chemical Co., Ltd. (Shanghai, China) and Meilun Biotech. Co., Ltd. (Dalian, China). The primary antibodies against GAPDH, ULK1, TNFα, FADD, FasL, Cytochrome C, Caspase‐3 and ‐8, Bcl‐2, mTOR and LC3 were purchased from Proteintech Co., Ltd. (Wuhan, China). The primary antibodies against p62, p‐ULK1, IL‐1β, RIP1, RIP3, MLKL, Caspase‐1 and NLRP3 were purchased from Cell Signaling Tech. (Danvers, MA, USA). The foetal bovine serum and DMEM culture were obtained from GIBCO (NY, USA). DAPI and trypsin were purchased from Keygen Biotech (Nanjing, China). The H9c2 cell lines were transfected with siRNA by Lipofectamine 2000 (Invitrogen, USA) according to the manufacture's protocol. Scramble siRNA Ribobio Co., Ltd. (Guangzhou, China) and human BNIP3‐specific siRNA (Santa Cruz, Cat# sc‐37451) were transfected with a final concentration of 10 nmol/L to the transfection culture for 6 hours or cultured overnight. The other reagents were of analytical grade and used directly unless stated otherwise.

### 2.2 Cell lines and culture

The rat cardiomyocyte H9c2 cell line was derived from rat embryonic heart tissue, respectively, and generally maintains their cardiomyocyte characteristics. The preparation of rat primary cardiocytes was performed according to a previously reported protocol.^19^ H9c2 and rat primary cardiocytes cells were cultured in DMEM supplemented with 10% FBS, streptomycin (100 mg/mL) and penicillin (100 U/mL) under a 5% CO_2_ atmosphere at 37°C. H9c2 cells were incubated for different times with aconitine (1.0 μmol/L), Enbrel (2.0 μmol/L) or the NLRP3 inhibitor MCC950 (2.0 μmol/L), which were dissolved in 0.1% DMSO alone or together in different combinations. The cell proliferation, apoptosis and reactive oxygen species (ROS) assays were performed according to the methods used in our previous reports, and the experimental procedures are described in the supplementary materials.

### 2.3 RNA extraction, next‐generation sequencing and bioinformatics analysis

Total RNA was extracted from aconitine‐ or normal saline‐treated H9c2 cells by using QIAGEN RNA extraction kits, and all experimental procedures were performed according to the manufacturer's protocol without additional modification. To guarantee a high‐quality RNA‐sequencing analysis, the quantity of extracted total RNA in each sample was above 2.0 μg, and a RIN value (RNA Integrity Number) ≥8.0 was used. NGS (next‐generation RNA sequencing) was performed on an Illumina HiSeq 4000 platform of Novogene Co., Ltd. (Beijing, China) to evaluate the differential gene expression profiles after aconitine incubation. The expression level of each gene was normalized by using the EdgeR method, and the transcriptomic differences between the control and aconitine‐treated groups were assessed by Student's *t* test. The criterion for significant DEGs (differentially expressed genes) was set to at least a 2‐fold change in gene expression, with an adjusted *P*‐value <.01. The DEGs were enriched by their KEGG (Kyoto Encyclopedia of Genes and Genomes) pathways with the GSEA (gene set enrichment analysis) method embedded in the clusterProfiler package.

### 2.4 Animal models

Sprague‐Dawley (SD) rats (8‐ to 10‐week‐old males) were purchased from Chengdu Dashuo Laboratory Animal Co., Ltd. (Chengdu, China). All animal experiments were performed in accordance with the Guide for the Care and Use of Laboratory Animals and with approval from the IACUC (Institutional Animal Care and Use Committee) and Ethics Committee of West China Hospital, Sichuan University. All animals were housed in sterilized filter‐top cages in an environment of controlled humidity at 22°C with a 12 hours to 12 h day‐to‐night cycle. Thirty rats were randomly divided into three groups. There were 10 rats in each group. The experimental group was orally administered with 1.0 mg/kg aconitine per day either alone or in combination with an intracardiac injection of 1 × 10^8^ TU BNIP3‐adenovirus. Rats in the control group received the same volume of normal saline. After 7 days of treatment, all rats were euthanized, and the heart tissues were sectioned and prepared for immunofluorescent and immunohistochemical analysis.

### 2.5 Immunohistochemical, immunofluorescence and TUNEL assays

The excised hearts were washed with saline solution, fixed in 10% formalin, and embedded in paraffin. Samples were sectioned (4‐6 μm thick) and stained with H&E and Masson's trichrome for microscope observation. Sections were incubated overnight at 4°C with the corresponding primary antibodies followed by incubation with the secondary antibody for 1 hour in the dark. Apoptotic cells were detected with a TUNEL staining kit. All slides were observed and imaged with a fluorescence microscope.

## 3 RESULTS

### 3.1 Aconitine inhibits myocardial cell proliferation and induces apoptosis

**Figure 1:**
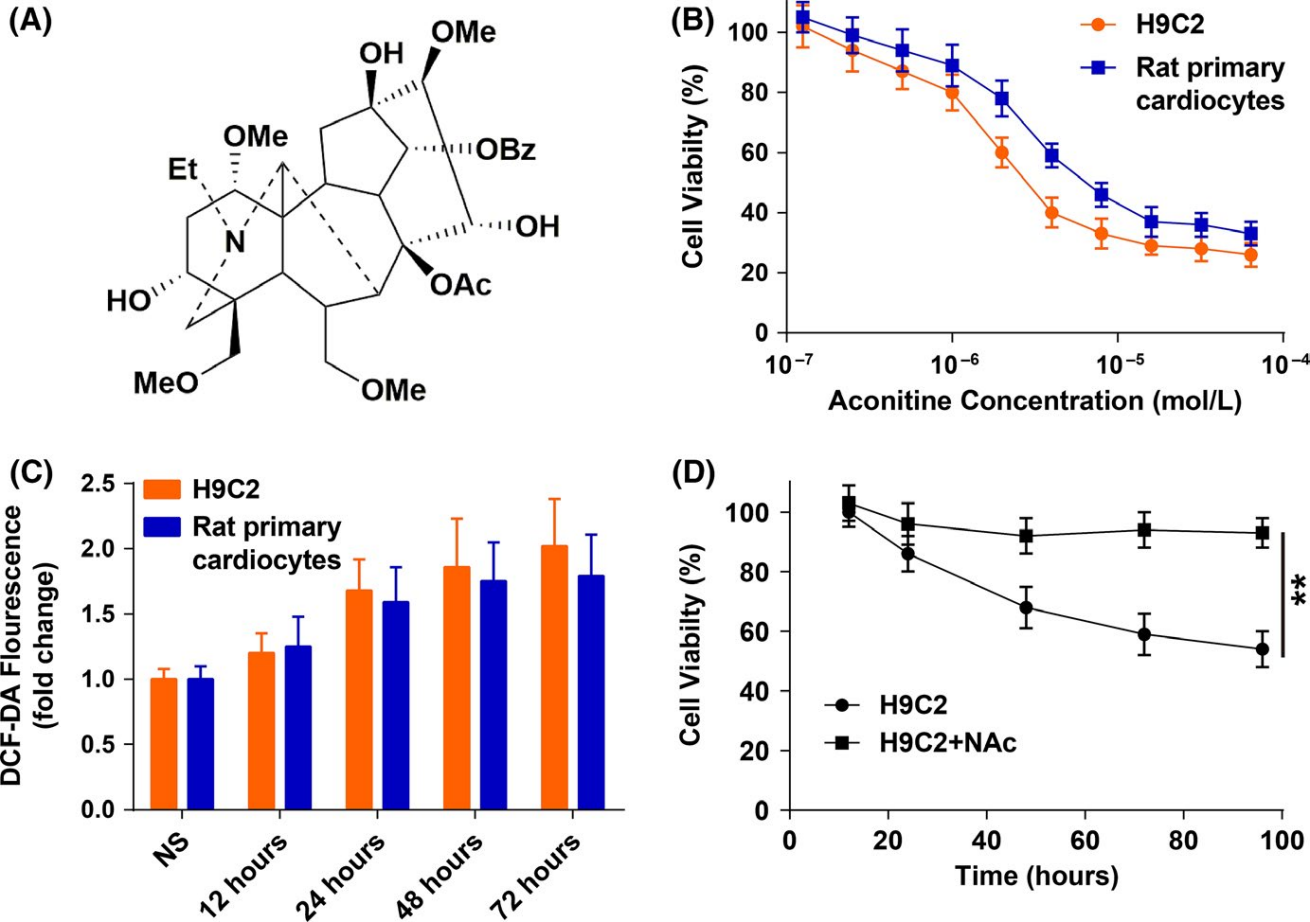
A, Chemical structure of aconitine; B, The viabilities of H9c2 cells and rat primary cardiocytes incubated with aconitine, as determined via the MTT method (*P < .05); C, The ROS levels of H9c2 cells and rat primary cardiocytes incubated with aconitine, as determined via the DCF‐DA method (*P < .05; **P < .01); D, The viability of H9c2 cells incubated with aconitine or aconitine plus N‐acetyl cysteine, as determined via the MTT method. The results are presented as the mean ± SD (n = 3) (**P < .01)

Aconitine's cytotoxicity (Figure 1A) on H9c2 rat cardiomyocyte and rat primary cardiocyte cell lines was assessed by using the MTT assay. The cell viabilities declined significantly after 24 hours of aconitine incubation in a dosage‐dependent manner, and H9c2 cell was more sensitive to aconitine incubation than that of rat primary cardiocytes (Figure 1B). ROS accumulation was also detected by DCF‐DA probes. As expected, the incubation of 1.0 μmol/L aconitine for more than 24 hours resulted in significant ROS accumulation with extended exposure time than the corresponding normal saline groups (Figure 1C). To further identify the relationship between ROS accumulation and aconitine's cytotoxicity, N‐acetyl cysteine (NAc) was added to aconitine‐treated H9c2 cells; aconitine's cytotoxicity was almost completely diminished by NAc (Figure 1D).^20^, ^21^, ^22^

**Figure 2:**
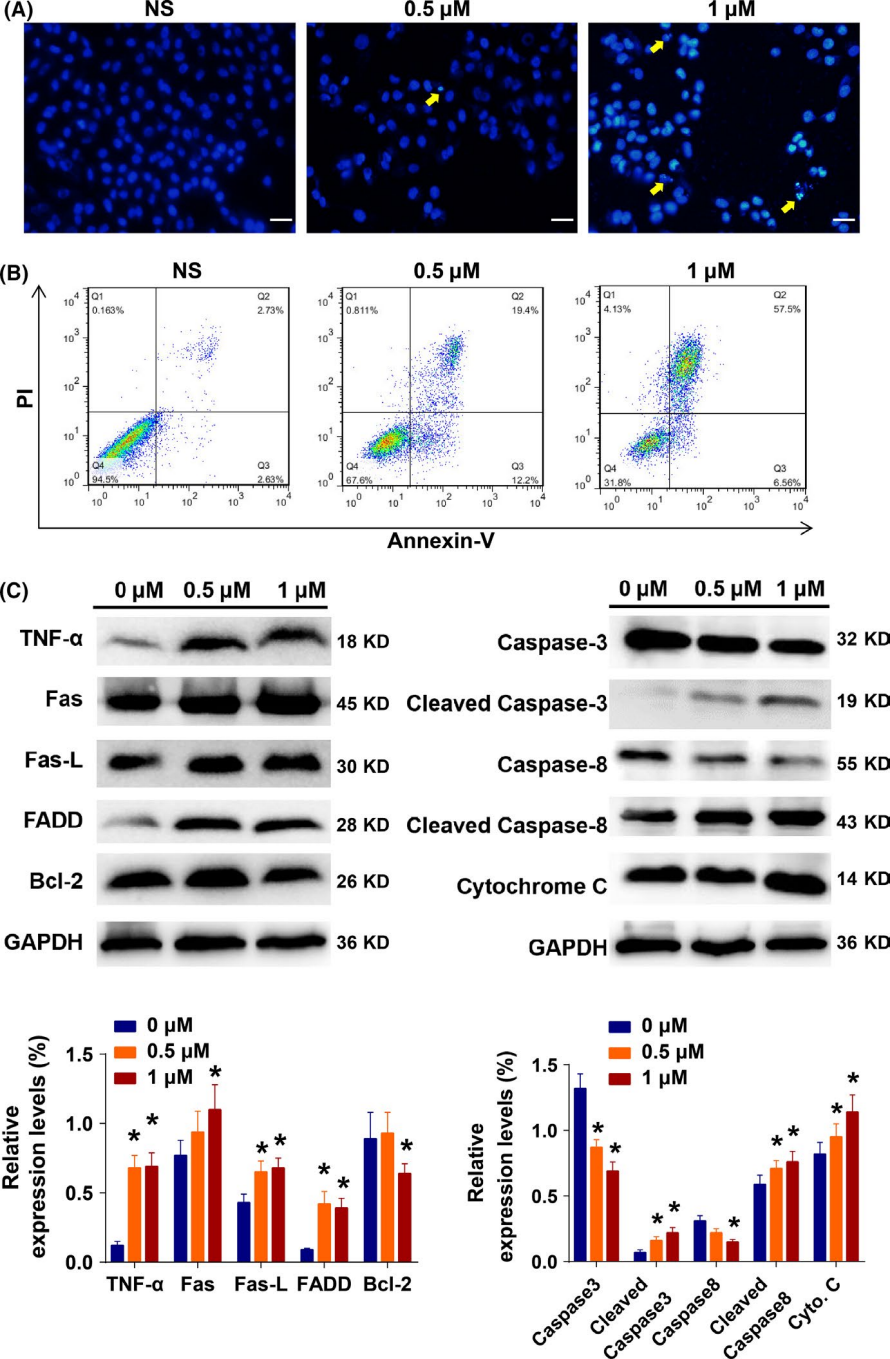
A, The apoptotic nuclei morphology changes of H9c2 cells after aconitine treatment with Hoechst 33258 staining, as determined by fluorescence microscopy; scale bar: 20 μm. B, The analysis of apoptotic H9c2 cells based on Annexin V/PI double staining by flow cytometry with aconitine incubation. C, Western blot analysis of apoptosis‐related proteins in total lysates of H9c2 cells after the indicated treatments (*P < .05 to 0 μmol/L group)

The apoptotic cell death of H9c2 cells induced by aconitine incubation was determined by Hoechst 33258 staining and Annexin V/PI double staining by flow cytometry. Apoptotic nuclei morphological changes were induced after aconitine incubation (Figure 2A) and significantly increased Annexin V‐ and/or PI‐positive cell counts (Figure 2B). Western blot analysis demonstrated that aconitine treatment (0.5 or 1.0 μmol/L, 24 hours) induced the expressions of the pro‐apoptotic factors TNFα, FADD, and cytochrome C and the cleavage of caspase‐3 and ‐8. The expression of the anti‐apoptotic indicator Bcl‐2 declined after 1.0 μmol/L aconitine incubation (Figure 2C). These findings confirmed that aconitine incubation triggers apoptotic cell death in H9c2 cardiomyocytes.^23^, ^24^

### 3.2 Bioinformatics analysis of aconitine‐treated H9c2 myocardial cells

**Figure 3:**
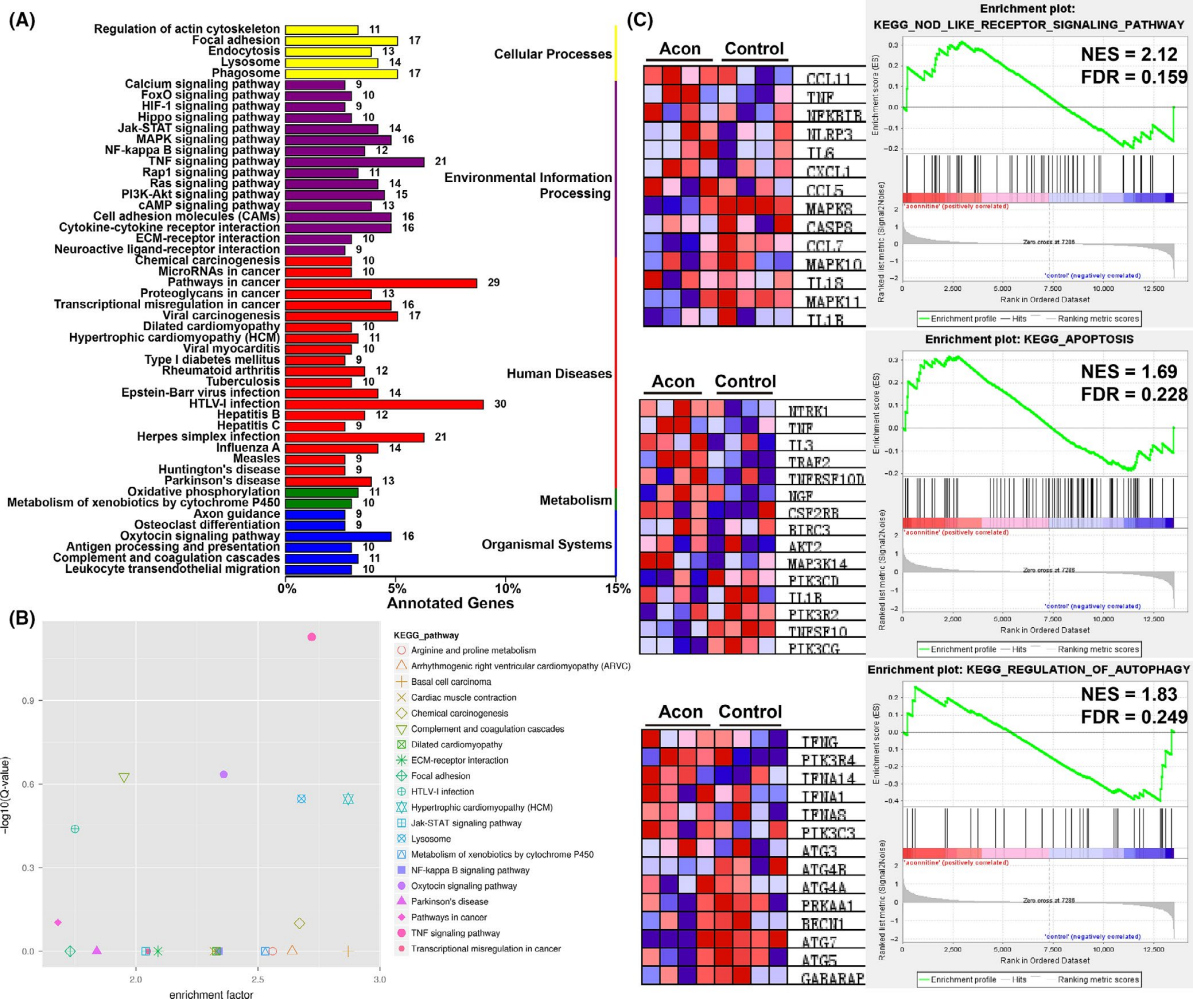
A, KEGG pathway enrichment of DEGs in H9c2 cells after aconitine incubation; B, Log_2_Q‐value vs enrichment factors plot of top‐ranked KEGG pathways; C, GSEA results of DEGs and representative genes in each pathway. NES: net enrichment scores; FDR: false discovery rate

Next, the gene expression profiles of H9c2 cells after aconitine treatment were determined by NGS of mRNA on an Illumina HiSeq 4000 platform.^25^, ^26^, ^27^, ^28^ There were 817 differentially expressed genes identified between the control and aconitine‐treated groups, including 503 upregulated and 314 downregulated genes (Figure S1). All DEGs were annotated and enriched based on their KEGG pathways,^29^, ^30^, ^31^, ^32^ and the Log_2_Q values and enrichment factors of the TNFα signalling, hypertrophic cardiomyopathy, lysosome and oxytocin signalling pathways were significantly enriched (Figure 3A,B). Moreover, the NLR (Nod‐like receptor) signalling pathway, apoptosis and regulation of autophagy pathways were top‐ranked pathways enriched by GSEA analysis (Figure 3C). Several inflammation‐related genes, such as TNF, IL‐1β and NLRP3, were common among these enriched pathways.^19^, ^33^, ^34^, ^35^

### 3.3 Both apoptosis and NLRP3 pathways were involved in aconitine's cytotoxicity

**Figure 4:**
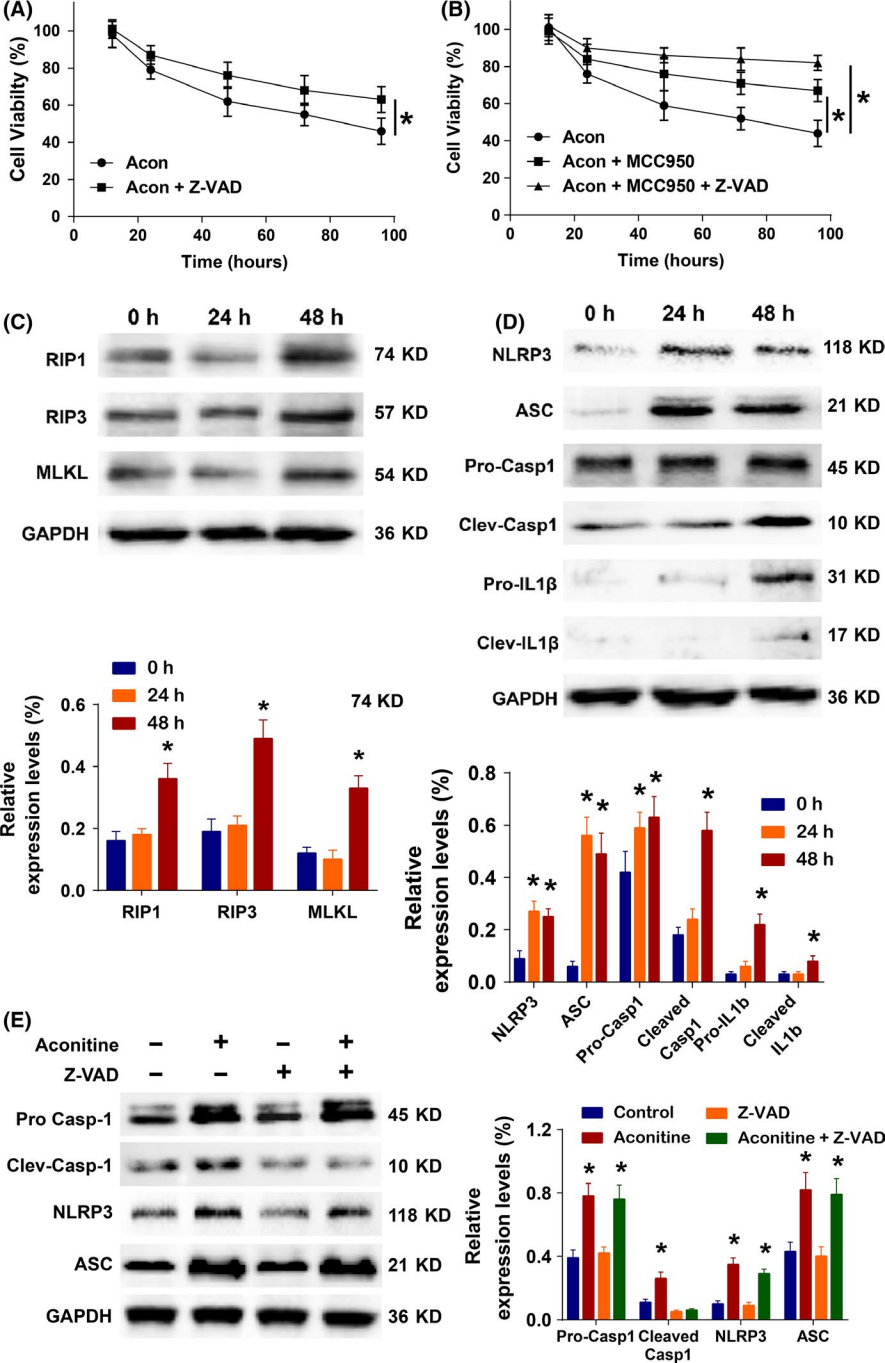
A‐B, The viability of H9c2 cells incubated with aconitine alone or a combination of aconitine and Z‐VAD, MCC950 or MCC950 plus Z‐VAD; C‐D, Western blot analysis of NLRP3 and RIPK pathway‐related proteins in total lysates of H9c2 cells after the indicated treatments (*P < .05)

To further study the role of apoptosis in aconitine‐induced cardiomyocyte damage, a pan‐caspase inhibitor, Z‐VAD, was added to the aconitine‐treated H9c2 cells and rat primary cardiomyocytes. The cell viability assay results demonstrated that aconitine's cytotoxicity was only partially reversed by Z‐VAD (Figure 4A and Figure S2A). There might be other cell death subroutines involved in aconitine's cytotoxicity (Figure 4B and Figure S2B). The detailed relationship and regulatory mechanisms between RIPKs and NLRP3 inflammatory pathways remain unclear.^36^ Some recent studies suggested that caspase‐8 activation could result in the formation of the NLRP3‐inflammasome and/or secretion of pro‐inflammatory interleukins via phosphorylated RIPKs or other unknown mechanisms.^37^

According to the Western blot analysis results, the RIPK pathway was stimulated by aconitine after 48 hours or more of incubation, as indicated by the increased expression of RIP1, RIP3 and MLKL (Figure 4C). The activation of the NLRP3 inflammasome was observed within 24 hours of aconitine incubation and was indicated by the increased expression of NLRP3 and ASC and the delayed activation of the pro‐inflammation indicator caspase‐1 and IL‐1β cleavage (Figure 4D). As expected, the addition of pan‐caspase inhibitor Z‐VAD could only suppress the cleavage of Caspase‐1 and did not affect the expressions of NLRP3 and ASC. Therefore, the NLRP3 inflammasome pathway (not caspase activation) was the main mechanism underlying the toxicity induced by aconitine.

**Figure 5:**
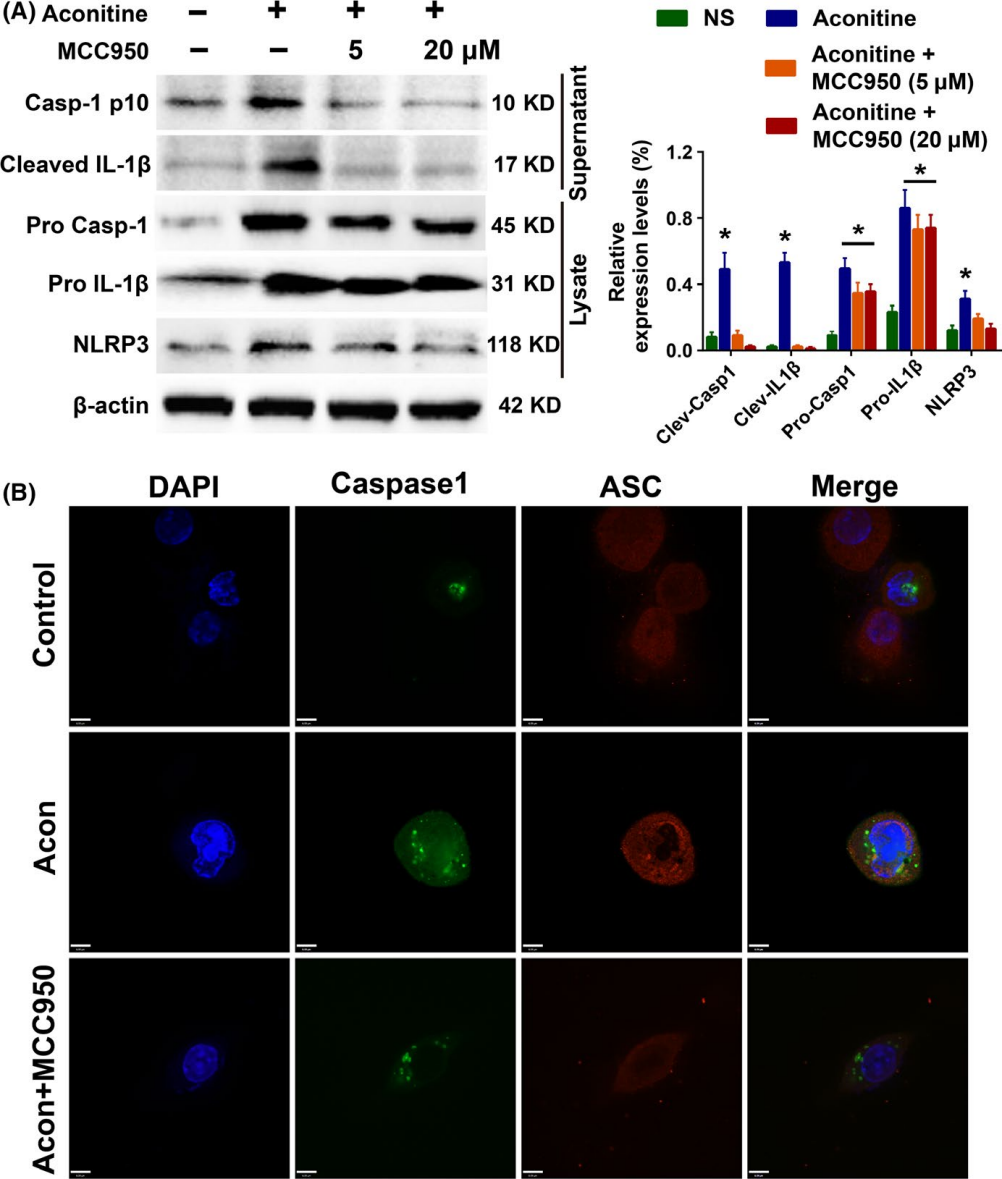
A, Western blot analysis of NLRP3 pathway activation after aconitine incubation for 24 h with or without the NLRP3 inhibitor MCC950; B, The subcellular location of NLRP3 and ASC used to detect NLRP3 inflammasome formation after aconitine incubation for 24 h with or without MCC950, scale bar: 6 μm

To further study the role of NLRP3 inflammasomes in aconitine‐induced cardiomyocyte damage, the NLRP3 inhibitor MCC950 was added to the aconitine‐treated H9c2 cells.^38^, ^39^, ^40^ The cleavage of pro‐Caspase‐1 and pro‐IL‐1β and expression of NLRP3 after aconitine incubation were severely suppressed by MCC950 rather than the transcript activation of Caspase‐1 and IL‐1β (Figure 5A). WB analysis of ASC, which is an NLRP3 partner protein required to assemble the NLRP3 inflammasome, failed several times after repeatedly changing the primary antibodies from different companies. The addition of aconitine potently stimulated the expression and assembly of NLRP3 inflammasome and Caspase‐1 activation. Moreover, the activation of NLRP3 inflammasome pathway could be reversed by NLRP3 inhibition of MCC950 in a dose‐dependent manner. Therefore, we performed an immunofluorescent assay to simultaneously visualize the protein expression changes of Caspase‐1 and ASC. As shown in Figure 5B, MCC950 significantly inhibited both Caspase‐1 and ASC expressions, as stimulated by aconitine incubation. Moreover, the colocalization of Caspase‐1 and ASC was also suppressed by the addition of MCC950 in aconitine‐treated H9c2 cells. NLRP3 inflammasome activation of aconitine was critical to the initiation of inflammatory cascade reactions.

### 3.4 A TNFα inhibitor ameliorates aconitine‐induced NLRP3 inflammasomes by activating mitophagy

**Figure 6:**
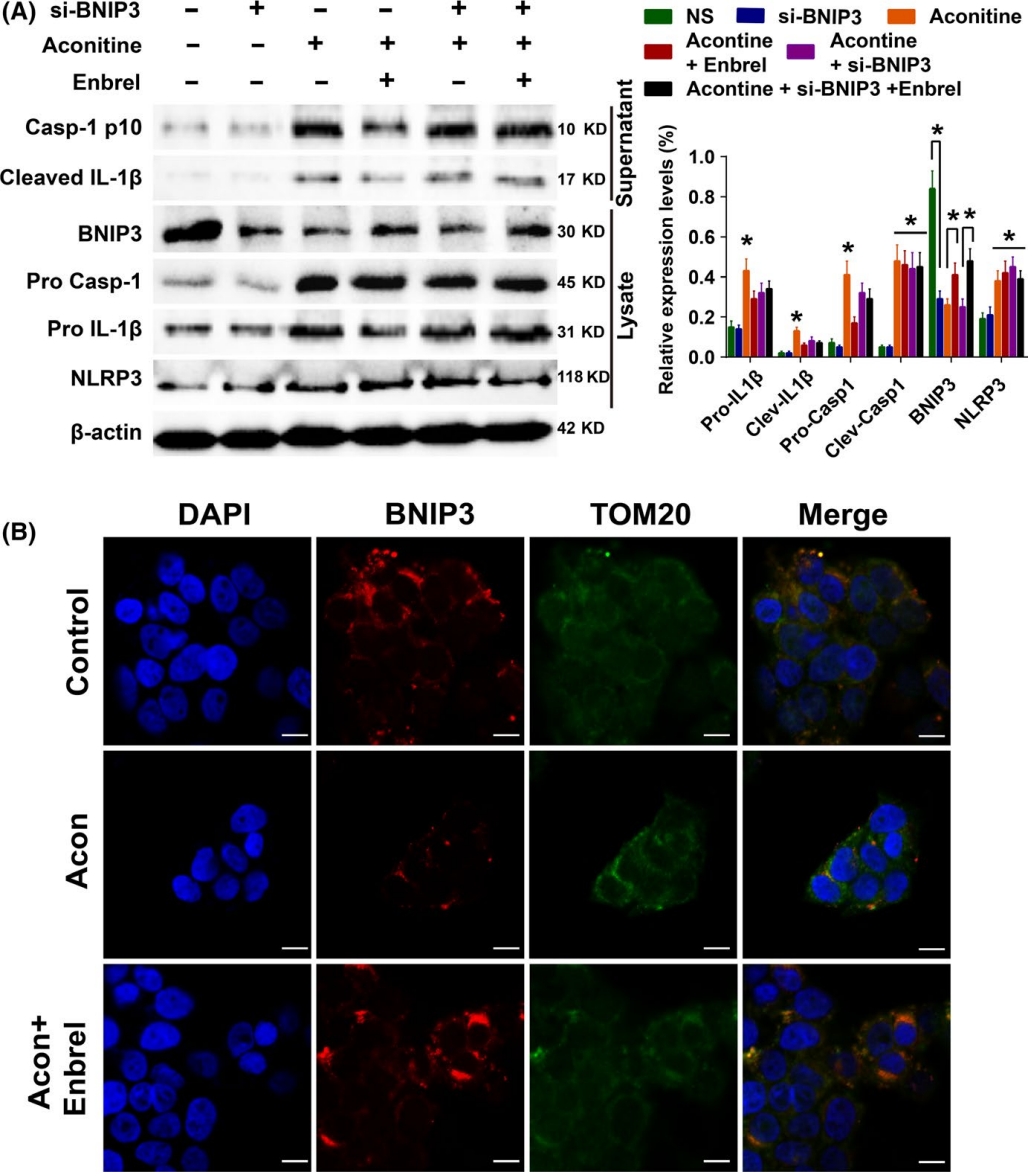
A, Western blot analysis of NLRP3 pathway activation after aconitine incubation for 24 h with or without the TNFα inhibitor Enbrel or BNIP3 shRNA. B, Immunofluorescence analysis of BNIP3 and TOM20 to detect mitophagy after aconitine incubation for 24 h with or without the TNFα inhibitor Enbrel, scale bar: 10 μm

To determine the effect and regulatory mechanism of TNFα in aconitine‐induced inflammation and mitophagy of H9c2 cardiomyocytes, cells were treated with aconitine at 2 μmol/L or with aconitine combined with the TNFα inhibitor Enbrel or BNIP3 shRNA for 24 hours. We considered that BNIP3 was a LC3‐interacted protein that played an important role in mitophagy, and the expression of BNIP3 was significantly declined after aconitine treatment. We speculated that BNIP3‐mediated mitophagy mighty involved in aconitine‐induced TNFα activation and inflammatory response in H9c2 cells. In Western blot analysis, the expression or cleavage of Caspase‐1 or IL‐1β was not affected by BNIP3 RNA interference alone (Figure 6A). Moreover, BNIP3 knockdown did not interfere with the aconitine‐induced activation of NLRP3, caspase‐1 or IL‐1β. The addition of TNF‐α inhibitor Enbrel could partially reverse the decreased expression of BNIP3 by aconitine or siBNIP3. Conversely, the addition of Enbrel significantly suppressed the Caspase‐1 expression and IL‐1β and Caspase‐1 cleavage. Furthermore, the inhibitory effects of Enbrel on aconitine‐induced Caspase‐1 and IL‐1β activation could be reversed by BNIP3 knockdown. Aconitine‐induced inflammation might be mediated by TNFα and BNIP3‐dependent mitophagy.^41^, ^42^, ^43^, ^44^, ^45^ To further identify the regulatory mechanism of TNFα and BNIP3 in aconitine‐induced inflammatory damage, an immunofluorescence assay was performed to visualize the expressions and subcellular locations of BNIP3 and TOM20, a mitochondrial membrane protein (Figure 6B). Aconitine did not change the expression of TOM20 but decreased the expression of BNIP3. The combination of Enbrel and aconitine strongly activated the expression of BNIP3 and promoted the colocalization of BNIP3 and TOM20. The aconitine‐induced cardiomyocyte damage might induce TNFα activation and then suppress BNIP3‐dependent mitophagy.

### 3.5 BNIP3‐dependent mitophagy alleviates the mitochondrial damage induced by aconitine

**Figure 7:**
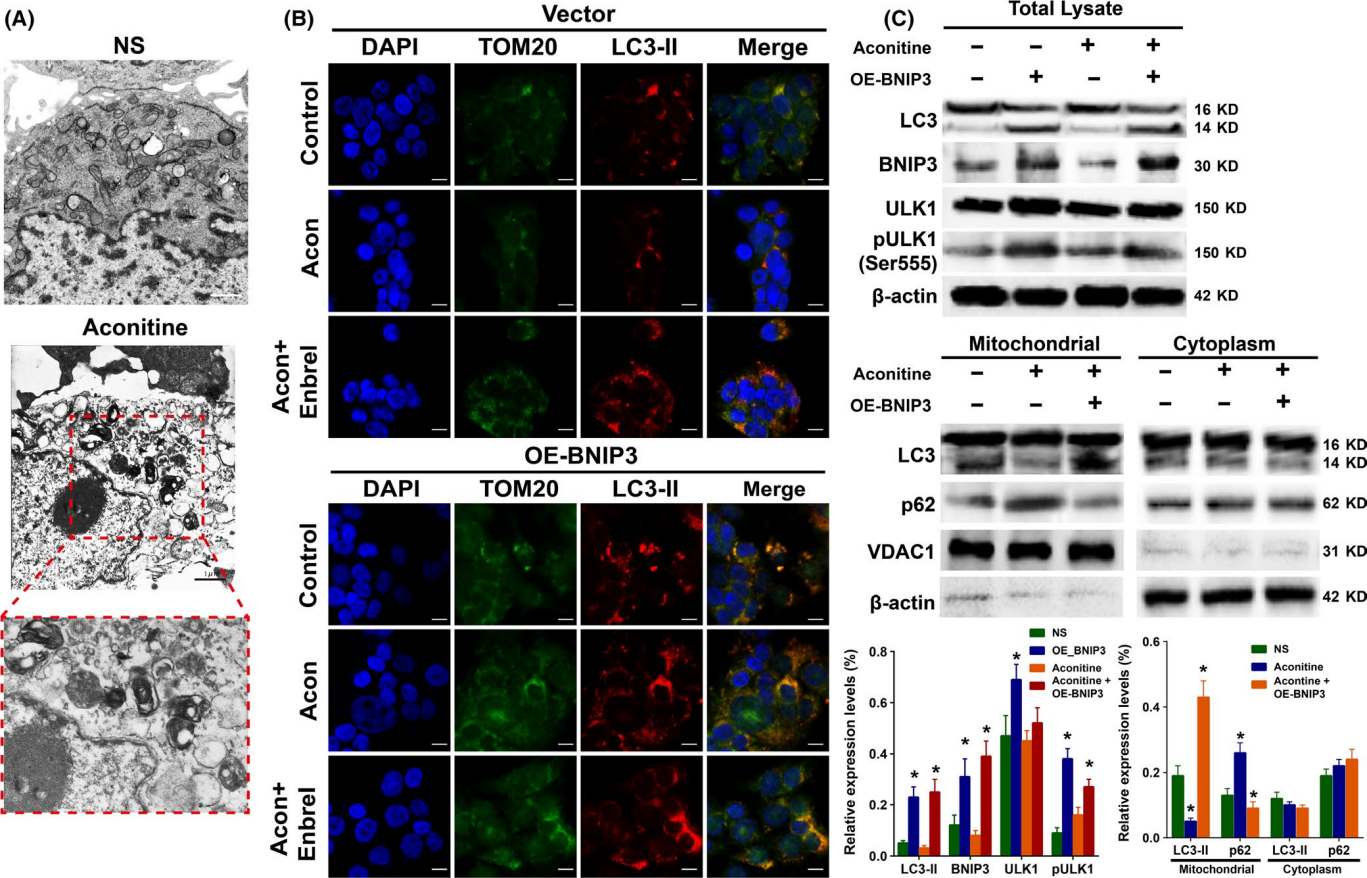
A, Morphological changes of mitochondria under TEM analysis, scale bar: 1 μm. B, Immunofluorescence of LC3 and TOM20 to detect mitophagy after aconitine incubation for 24 h with or without the TNFα inhibitor Enbrel under transfection of null vector or BNIP3‐overexpressed plasmid, scale bar: 10 μm. C, Western blot analysis of the autophagy‐related proteins LC3 and p62 after aconitine incubation for 24 h with or without pretransfected BNIP3‐overexpressed plasmid

The aconitine‐induced mitochondrial damages on H9c2 cells were detected by transmission electronic microscope (TEM) analysis and shown in Figure 7A. Compared with the control group, there were markedly swollen and damaged mitochondria observed in the cytoplasm. To better recognize the influences of BNIP3 and TNFα on protective mitophagy in H9c2 cells with aconitine incubation,^46^, ^47^, ^48^, ^49^ TOM20 and LC3 immunofluorescence assays were performed in H9c2 cells transfected with null vector or BNIP3 plasmid, with or without a combination of aconitine or aconitine plus Enbrel treatment, respectively.^50^, ^51^, ^52^, ^53^ As expected, BNIP3 overexpression and the TNFα inhibitor significantly stimulated LC3 expression, accumulation and colocalization to mitochondria in cells treated with aconitine (Figure 7B).

According to the WB results shown in Figure 7C, overexpression of BNIP3 activated LC3 cleavage and ULK1 phosphorylation, which suggested that autophagy was activated. Moreover, the mitochondria and cytoplasm of H9c2 cells were separated, and LC3 and p62 protein expressions were determined by WB analysis. LC3 cleavage and p62 degradation, either inhibited by aconitine or activated by BNIP3 overexpression, occurred in the mitochondrion but not in the cytoplasm. Aconitine‐induced cardiomyocyte mitochondrial damage depended on the activation TNFα and inhibition of BNIP3‐mediated mitophagy.

### 3.6 Intracardiac injection of BNIP3‐overexpression adenovirus decreased aconitine‐induced cardiotoxicity

**Figure 8:**
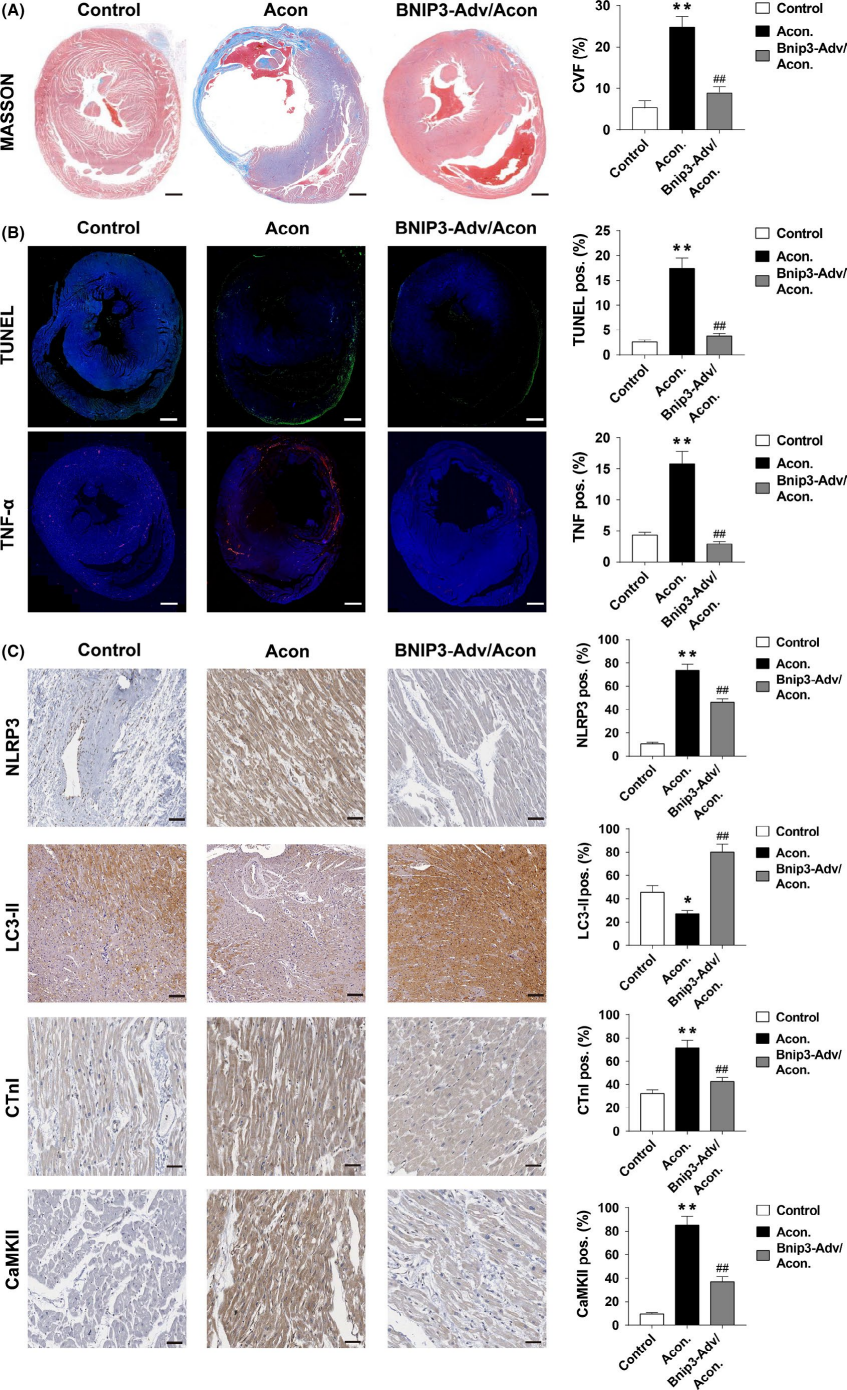
A, Masson's trichrome staining of myocardial tissue sections of control, aconitine treated and aconitine plus BNIP3‐OE rats, scale bar: 1 mm. B, immunofluorescent staining of TUNEL and TNFα in myocardial tissue sections of control, aconitine treated and aconitine plus BNIP3‐OE rats, scale bar: 1 mm. C, The immunohistochemistry analysis of NLRP3, LC3, CTnI and CaMKII in myocardial tissue sections of control, aconitine treated and aconitine plus BNIP3‐OE‐treated rats, scale bar: 50 μm. *P < .05 vs control. **P < .01 vs control. ##*P* < .01 to aconitine‐treated group

IHC staining of BNIP3 expression in the heart tissues of rats in the null vector group and the BNIP3‐OE adenovirus group displayed the significant overexpression of BNIP3 in the BNIP3‐OE adenovirus group (Figure S3). Masson's trichrome staining was performed to evaluate the degree of cardiac inflammatory fibrosis. Aconitine‐treated rats developed higher cardiac fibrosis, which was significantly prevented by the addition of BNIP3‐overexpression adenovirus (Figure 8A). BNIP3‐OE reversed aconitine‐induced activation of TNFα, NLRP3, LC3 and cardiac apoptosis as assessed by TUNEL staining (Figure 8B,C). The IHC staining of myocardial damage marker CTnI (Cardiac Troponin I) and CaMKII (Ca2+/calmodulin‐dependent protein kinase II) indicated that the overexpression of BNIP3 obviously suppressed aconitine‐induced myocardial damage. As observed in vitro, BNIP3 overexpression potently reversed aconitine‐induced cardiotoxicity by enhancing mitophagy.

## 4 DISCUSSION

*Aconitum* species are widely used as TCMs for the treatment of various diseases. Thus, the cardiotoxicity of aconitine and its derivatives and/or metabolites have potential risks, but details of the underlying cardiotoxicity mechanism have not been elucidated. Few reports indicated that aconitine induces oxidative stress stimuli in myocardial cells via ROS accumulation, thereby inducing lipid peroxidation and inflammatory cytokine release.^54^, ^55^, ^56^ Aconitine‐induced ROS accumulation results in the activation of the PPAR activator PGC‑1α and in an apoptotic response to oxidative stress damage.^20^

In our study, aconitine suppressed the proliferation and induced oxidative damage in H9c2 cell line and primary rat cardiomyocytes. The addition of antioxidant NAc potently inhibited aconitine's cytotoxicity, which suggested that oxidative damage played a vital role in the toxic mechanism of aconitine. The FCM and WB analysis results exhibited that aconitine‐induced mitochondria apoptosis and inflammatory response in H9c2 cells. The addition of caspase inhibitor Z‐VAD only partially reversed aconitine's cytotoxicity in both H9c2 and primary rat cardiomyocytes, and the combination of NLRP3 inhibitor MCC950 and caspase inhibitor Z‐VAD almost totally suppressed the proliferation inhibitory capacity of aconitine. Based on the bioinformatics analysis of significantly enriched pathways in DEGs after aconitine incubation, NLRP3, apoptosis and autophagy pathways were highly enriched in the aconitine‐treated group. Moreover, mitophagy was suppressed after aconitine incubation, as detected by TEM, immunofluorescence and Western blot analysis of the H9c2 cells. BNIP3 acted as an LC3 interacted protein in mitophagy and then mediated the autophagic degradation of damaged mitochondrion.^57^, ^58^, ^59^ We identified the beneficial effects of TNFα inhibition and BNIP3‐mediated mitophagy on the myocardial dysfunction of aconitine treatment. The inhibition of mitophagy and mitochondrial damage caused by aconitine might be crucial in the development of aconitine‐induced myocardial inflammation and apoptosis. ULK1 phosphorylation, LC3 cleavage and p62 degradation in the mitochondria were suppressed after aconitine incubation, and the canonical NLRP3 inflammasome and apoptosis pathways were activated by aconitine. The TNFα inhibitor Enbrel and the BNIP3‐OE plasmid could activate protective mitophagy in aconitine‐treated myocardial cells. Intriguingly, the mitophagy activated by Enbrel or BNIP3‐OE coincided with a decline in NLRP3 signalling cascades. Recently, Ma et al reported the reduction of aconitine cardiotoxicity by sweroside, which destabilized and disrupted the mitochondrial membrane potential and dysregulated autophagic proteins. However, this is the first report on the alleviation of aconitine cardiotoxicity through the activation of BNIP3‐mediated mitophagy and inhibition of TNFα signals in myocardial cells. The relationship among inflammation, apoptosis and cardiotoxicity of aconitine is complicated, according to the most recently published literature. Huang et al reported that aconitine‐containing TCM extractions activated the PI3K/Akt/mTOR signalling pathway, as well as TGFβ1 and apoptosis. Furthermore, mitochondrial apoptosis, endoplasmic reticulum stress, and pro‐inflammatory and oxidative stress responses also participated in the myocardial injury regulation network of aconitine treatment. Although the direct molecular mechanisms underlying the way by which BNIP3‐mediated mitophagy alleviated aconitine‐induced myocardial injury were not fully identified, our findings lay a foundation for the use of TNFα inhibitor and BNIP3 for aconitine‐induced cardiac toxicity prevention and therapy, which should require further investigation.

In conclusion, we have investigated the molecular mechanisms and prevention/therapeutic strategy of the cardiac toxic natural product aconitine. The BNIP3‐mediated mitophagy potently alleviates myocardial injuries of aconitine both in vitro and in vivo. The aconitine cytotoxicity in cardiomyocytes is dependent on the activation of the TNFα and NLRP3 inflammasome pathways, which may provide novel insights into the prevention of aconitine‐related toxicity.

## CONFLICT OF INTEREST

The authors declare that they have no conflict of interest.

## AUTHORS’ CONTRIBUTIONS

FP, BH and GH involved in conceptualization; NZ and FP involved in data curation; NZ and FP formally analysed; BH and GH involved in funding acquisition; BH and GH investigated; NZ and FP involved in methodology; BH and GH administrated the project; CW, CP and WH resourced; FP and CW involved in software; BH and GH supervised; NZ, FP and GH wrote the original draft; FP, BH and GH reviewed and edited the writing. All authors read and approved the final manuscript.

## ETHICAL APPROVAL

All procedures involving animals were performed in compliance with guidelines of the Chengdu University of Traditional Chinese Medicine.

## Data Availability

The data that support the findings of this study are available from the corresponding author upon reasonable request.
