# Supplementary material for: Aconitine induces cardiomyocyte damage by mitigating BNIP3‐dependent mitophagy and the TNFα‐NLRP3 signalling axis
Source: Cell Prolif. 2019 Oct 27;53(1):e12701. doi: 10.1111/cpr.12701 (PMC6985658; doi:10.1111/cpr.12701)
Supplement: Supplementary file 1 [file CPR-53-e12701-s001.docx]

**Aconitine induces cardiocyte damage by mitigating BNIP3-dependent mitophagy and TNFα-NLRP3 signaling axis**

Fu Peng^a^, Nan Zhang^a^, Chunting Wang^a^, Xiaoyun Wang^a^, Wei Huang^b^, Cheng Peng^b^, Gu He^a,^* and Bo Han^b,^*

^a^ *West China School of Pharmacy, and State Key Laboratory of Biotherapy, West China Hospital, Sichuan University, Chengdu 610041, China.*

^b^ *Key Laboratory of Southwestern Chinese Medicine Resources, School of Pharmacy, Chengdu University of Traditional Chinese Medicine, Chengdu 611137, China.*

* Corresponding authors.

E-mail address: hegu@scu.edu.cn (Gu He, +86-28-85503817)

E-mail address: hanbo@cdutcm.edu.cn (Bo Han, +86-28-61800231)

**Supplementary Materials**

**Isolation and culture of rat primary cardiocytes**

The isolation and culture of rat primary cardiocytes were according to the literatures’ method. In brief, six to right-week-old male SD rats were anesthetized with isofluorane, heparinized, sacrificed, and cardiac myocytes were then isolated. The heart was quickly removed from the chest and retrogradly perfused through the aorta with a calcium-free HEPES-buffered solution. The heart was then enzymatically digested with type 2 collagenase, protease XIV and the calcium concentration was steadily increased to 200 μM. The heart was minced and digested further with gentle agitation at 37 °C and trituration over 10 min. Finally, the isolated cardiocytes were filtered and washed twice with a buffer containing 0.1% BSA and 1.25 mM calcium chloride prior to plating. Cardiocytes were cultured in M199 medium (Gibco) supplemented with 10% fetal bovine serum, penicillin/streptomycin, L-carnitine, creatine, and taurine.

**Cell viability assay**

The rat primary cardiocytes or H9C2 Cells were seeded at 10^4^ cells/well into 96-well plates and incubated overnight, then treated with different concentrations of aconitine for desired time. The medium of each well was replaced by 200 µL fresh medium and 20 µL freshly prepared MTT (5 mg/mL in PBS) and the plate was incubated in the dark at 37 ℃ for 4 h. The culture medium was then removed and 200 µL DMSO was added to each well to dissolve formazan crystals, and absorbance was read at 570 nm using a microplate reader. All assays were performed in triplicate.

**Western blot analysis**

The protein extracts from H9C2 cells or cardiac muscle tissue were separated by sodium dodecyl sulfate-polyacrylamide gel electrophoresis (SDS-PAGE) and transferred onto a polyvinylidene difluoride (PVDF) membrane. Membranes were blocked with TBST containing 5% fat free milk for 1h and incubated with the different primary antibodies as indicated overnight at 4 °C. The membranes were then incubated with horseradish peroxidase conjugated secondary antibodies for 1h and visualized by the enhanced chemiluminescence system. The quantitative analyses were conducted by ImageJ software.

**Flow cytometry**

Flow cytometry was used to determine the apoptosis rate among cells. Apoptotic cells rate was detected by the combined application of Annexin V-FITC and PI. Cells were washed twice and adjusted to a concentration of 1 × 10^6^ cells/mL with cold Hanks buffer. Then, the cells were incubated with Annexin V-FITC and PI for 15 min at room temperature in the dark. Finally, the apoptotic cell rates were determined using flow cytometry and data were analyzed by FlowJo software. Each experiment was performed at least in triplicate.

**Transmission electron microscopy**

H9C2 cells treated with 1.0 μM aconitine for 24 h were collected and washed with 4℃ PBS, followed by fixing with 4% glutaraldehyde in 0.1 M sodium cacodylate for 2 h. Next, the samples were post-fixed with 1% OsO_4_ for 1.5 h, washed, dehydrated and embedded in Epon-Araldite resin. Ultrathin sections (80 nm) cut in a Reichert ultramicrotome were stained with 3% aqueous uranyl acetate for 1 h, and counterstained with 0.3% lead citrate. Samples were examined by transmission electron microscopy (HT7700, Hitachi, Japan).


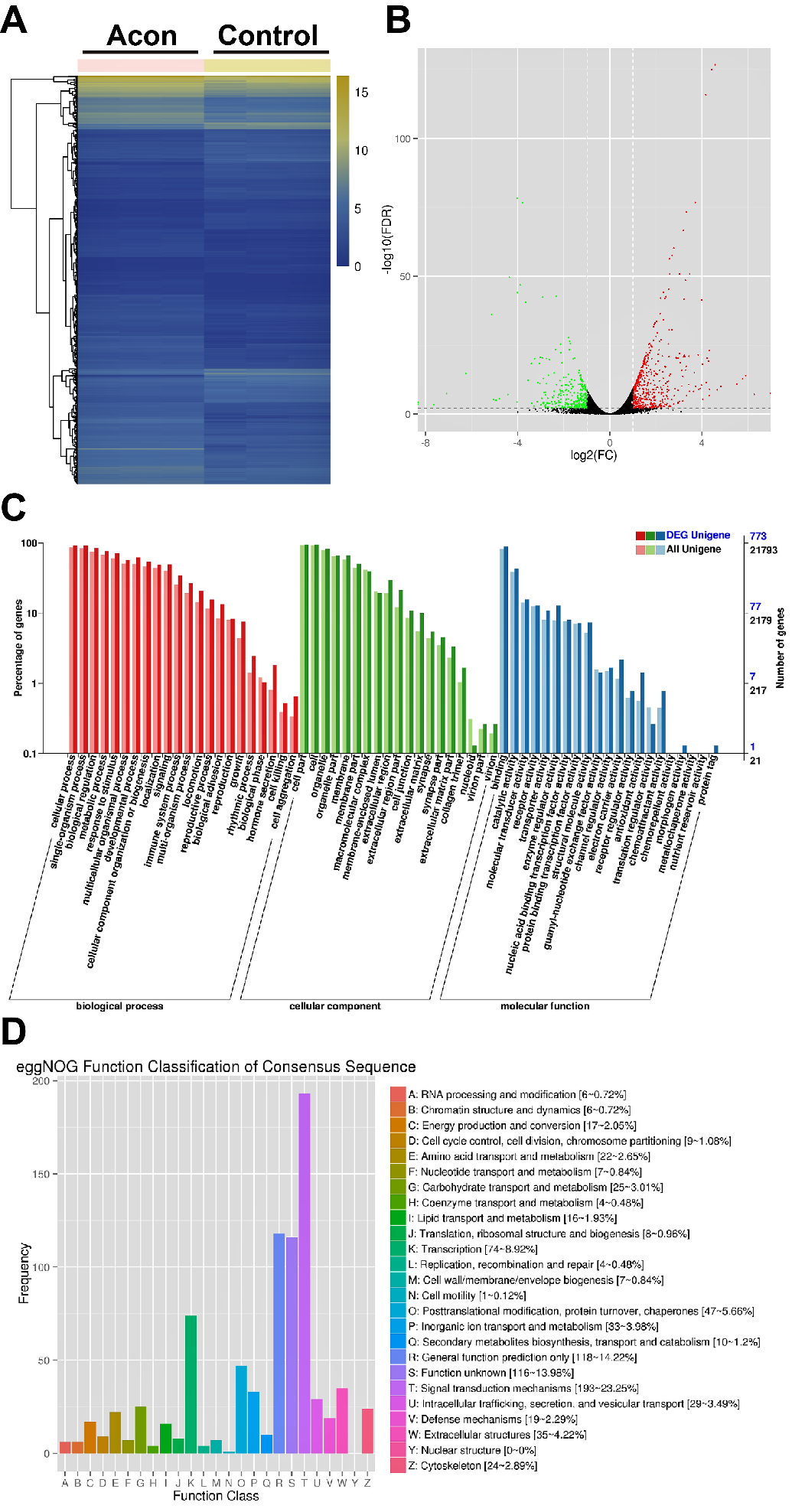


**Figure S1.** Bioinformatics analysis of RNA-seq results of H9C2 cells treated by aconitine. (A) the cluster of all genes by Hierarchical clustering method; (B) Volcano plot of all detected genes by using the Log_2_FC and –Log_10_FDR as cut-off, red denotes ‘upregulated genes’ whereas green denotes ‘downregulated genes’ on the basis of |FC (fold change)| >2.0 & a corrected P-value of <0.01; (C) Gene Ontology (GO) annotation and enrichment of differential expressed genes; (D) eggNOG (evolutionary genealogy of genes: Non-supervised Orthologous Groups) analysis of the significantly enriched pathways in all differential expressed genes.


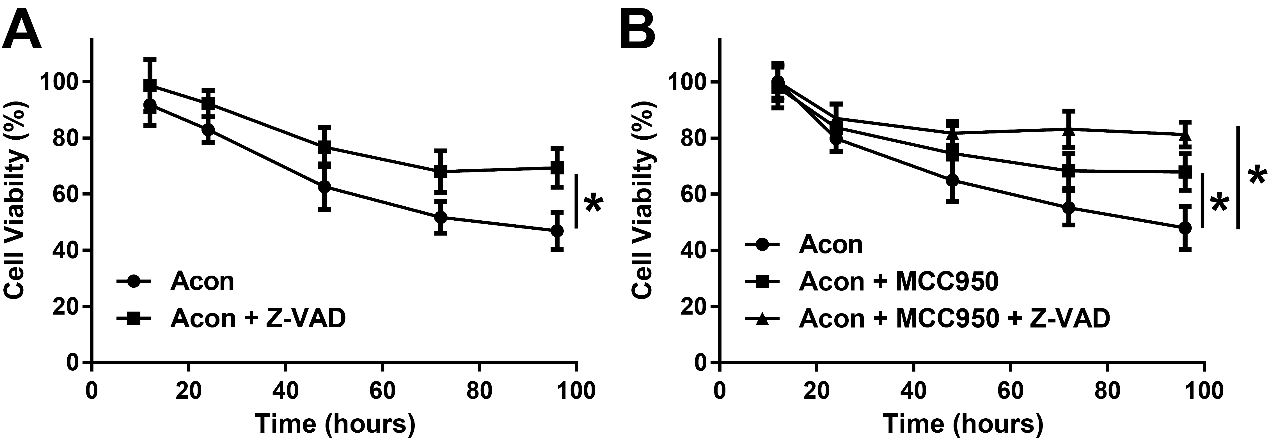


**Figure S2**. The viability of rat primary cardiomyocytes incubated with aconitine alone or a combination of aconitine and Z-VAD (A), MCC950 or MCC950 plus Z-VAD (B).


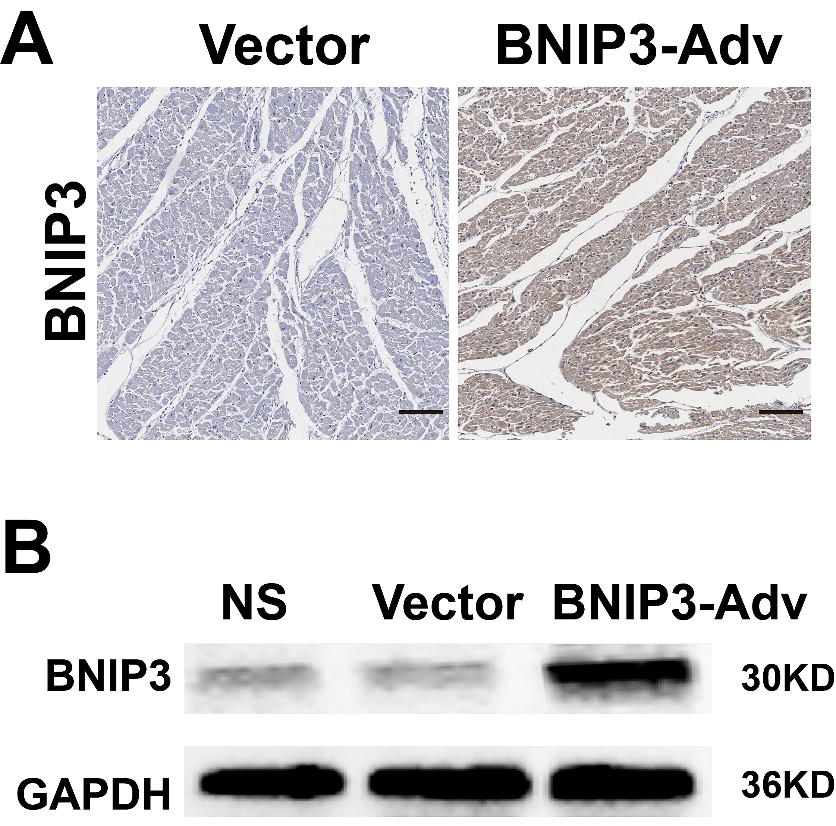


**Figure S3.** Identification of BNIP3 expression levels in rat heart tissue by IHC (A) and WB analysis (B) after intracardiac injection of BNIP3-overexpressed adenovirus, scale bar: 8.0 μm.
